# Supplementary material for: A model of population dynamics with complex household structure and mobility: implications for transmission and control of communicable diseases
Source: PeerJ. 2020 Nov 3;8:e10203. doi: 10.7717/peerj.10203 (PMC7646303; doi:10.7717/peerj.10203)
Supplement: Supplemental Information 1 [file peerj-08-10203-s001.pdf]

Supplementary material

A model of population dynamics with complex household structure and mobility: implications for transmission and control of communicable diseases

Rebecca H. Chisholm, Bradley Crammond, Yue Wu, Asha C. Bowen, Patricia T. Campbell, Steven Y. C. Tong, Jodie McVernon, Nicholas Geard

A Details of an extended model accounting for temporary-visitors

In our extended model we account for a third type of mobility due to regular events: funerals, and sporting matches. These events can be associated with the influx of large numbers of people into communities who stay for a short amount of time (a number of days).

We assume funerals occur after each death in the community, and that sporting matches occur every 7 days (e.g., weekend football matches). We assume that each visitor stays in one dwelling (selected uniformly at random) for the entire duration of their stay, and that their age, infection and immunity status are determined in the same way as for inter-community mobility. The duration of stay for sporting matches and funerals are assumed to be exponentially distributed with mean values set to between $[1, 2]$ and $[1, 7]$ days, respectively. The number of visitors for sporting matches and funerals are assumed to be Poisson distributed with mean values ranging between $[50, 100]$ and $[10, 50]$, respectively.

With these assumptions, the occupancy distributions of dwellings show similar characteristics to those from the original model, with clear steps corresponding to different resident types (compare figure 2 and S1). However, there are some dwellings with an increased number of unique residents that stay for a low number of days, due to event visitors.

B Supplementary tables

Table S1: Statistics from model scenarios of unmitigated outbreaks, including percentage of simulations that led to an outbreak (take off %), and the median (50%CIs) of the outbreak duration and final size.

Scenario	Dwelling occupancy	N	Take off (%)	Duration (days)	Final size
Higher q	fluid	2500	77.2	78 (68,90)	2225 (2011,2353)
Higher q	stable	2500	74.9	90 (78,105)	2068 (183,2236)
Lower $\hat{q}$, Less crowding	fluid	2500	32.6	138 (105,178)	885 (305,1340)
Lower $\hat{q}$ Less crowding	stable	2500	29.1	145 (102,182)	710 (154,1081)
Higher q, Less crowding	fluid	2500	60.8	108 (94,130)	1643(1304,1899)
Higher q Less crowding	stable	2500	60.3	115 (99,136)	1445 (1129,1695)
Baseline	fluid	500	51.3	82 (69,98)	339 (248,392)
Baseline	stable	500	49.3	87 (72,105)	301 (206,356)
Lower $\hat{q}$	fluid	500	35.9	89 (67,113)	208 (108,283)
Lower $\hat{q}$	stable	500	30.2	89 (66,107)	190 (88,260)
Less crowding	fluid	500	33.7	90 (69,110)	210 (105,283)
Less crowding	stable	500	32.5	89 (67,111)	167 (66,234)
Higher q	fluid	500	76	63 (56,75)	435 (385,462)
Higher q	stable	500	72.1	70 (60,81)	401 (357,436)
Lower $\hat{q}$, Less crowding	fluid	500	37.6	89 (69,110)	197 (98,286)
Lower $\hat{q}$ Less crowding	stable	500	31.5	88 (64,112)	174 (89,242)
Higher q, Less crowding	fluid	500	59.4	77 (66,91)	340 (272,389)
Higher q Less crowding	stable	500	58.5	80 (69,95)	299 (242,349)

Table S2: Statistics from additional model scenarios of mitigated outbreaks in communities of size 2500, including the percentage reduction in the median value of the outbreak duration and final size compared to the equivalent unmitigated scenarios, for 100% and 50% effective interventions.

Scenario	Dwelling occupancy	Median duration reduction (%) with intervention effect		Median final size reduction (%) with intervention effect	
		100%	50%	100%	50%
Higher q	fluid	22	-22	84	28
Higher q	stable	16	-17	90	30
Lower $\hat{q}$, Less crowding	fluid	30	8	70	42
Lower $\hat{q}$ Less crowding	stable	57	12	89	44
Higher q, Less crowding	fluid	19	-1	68	29
Higher q Less crowding	stable	14	-1	77	33

Table S3: Statistics from model scenarios of mitigated outbreaks in communities of size 500, including the percentage reduction in the median value of the outbreak duration and final size compared to the equivalent unmitigated scenarios, for 100% and 50% effective interventions.

Scenario	Dwelling occupancy	Median duration reduction (%) with intervention effect		Median final size reduction (%) with intervention effect	
		100%	50%	100%	50%
Baseline	fluid	45	2	83	45
Baseline	stable	57	9	92	52
Lower $\hat{q}$	fluid	53	27	81	56
Lower $\hat{q}$	stable	56	29	86	58
Less crowding	fluid	40	20	71	40
Less crowding	stable	52	21	82	52
Higher q	fluid	33	-10	82	26
Higher q	stable	37	-7	88	30
Lower $\hat{q}$, Less crowding	fluid	37	13	69	35
Lower $\hat{q}$ Less crowding	stable	45	24	76	49
Higher q, Less crowding	fluid	32	4	67	29
Higher q Less crowding	stable	33	4	75	27

C Supplementary figures

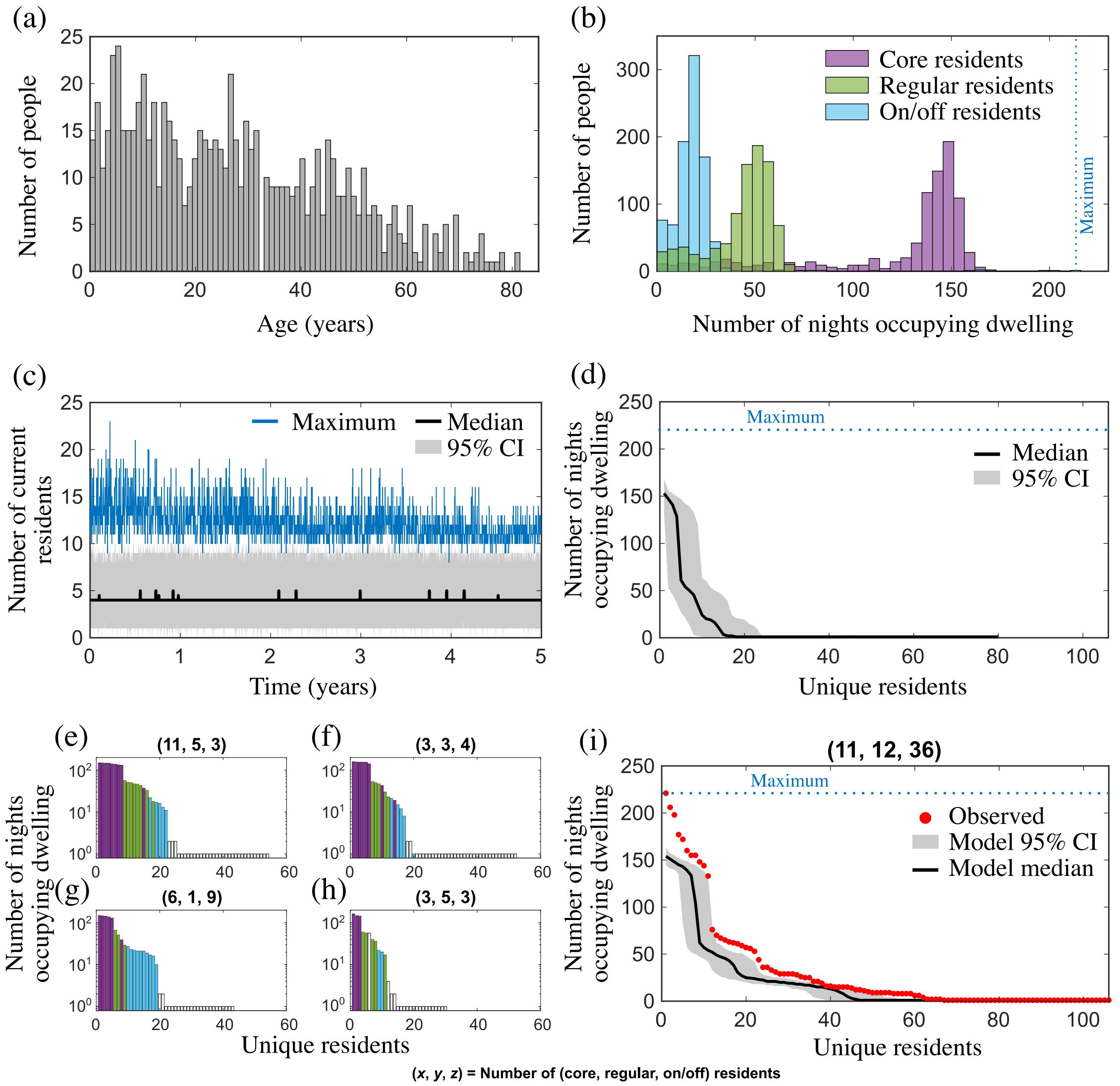

Figure S1: Extended population and mobility model with regular event migration. All simulations presented here were initialised with the same population as those presented in Figure 2 in the main text. Population and mobility model outputs from (a–h) one simulation; and (i) 100 simulations, for a community with similar characteristics to Yuendumu (NT, Australia). (a) Age distribution of the population (of permanent residents only) in years; (b) Number of nights that core (purple), regular (green) and on/off (blue) residents occupied their core, regular and on/off dwellings, respectively, over 221 nights; (c) The distribution of the number of current residents in each dwelling over 5 years, showing the median (black line), maximum (blue line), and 95%CI (grey shading); (d) The distribution of cumulative dwelling occupancy over 221 nights for all dwellings. The nth unit of the horizontal axis represents the nth-most regular occupant of a dwelling, and the vertical axis represents the median (black line) and 95%CI (grey shading) for the cumulative number of nights stayed by this occupant; (e–h) The cumulative dwelling occupancy over 221 nights for four exemplar dwellings. Each bar represents a unique individual (coloured according to resident type: core, purple; regular, green; on/off, blue; white, sporadic visitor) who stayed at the dwelling for at least one night, and the height of the bar represents the cumulative number of nights the individual was present (note, a log scale is used). Individuals are shown in order of decreasing occupancy, and the title of each subplot shows the number of (core, regular, on/off) residents for that dwelling at the end of the simulation. Note that the same dwellings are presented here as in Figure 2e–h in the main text which allows them to have the same number of residents of each type, as indicated in the subplot tiles; (i) Observed occupancy (red dots) vs model occupancy (median and 95%CI from 100 simulations) for a dwelling with 11 core, 12 regular, and 36 on/off residents.

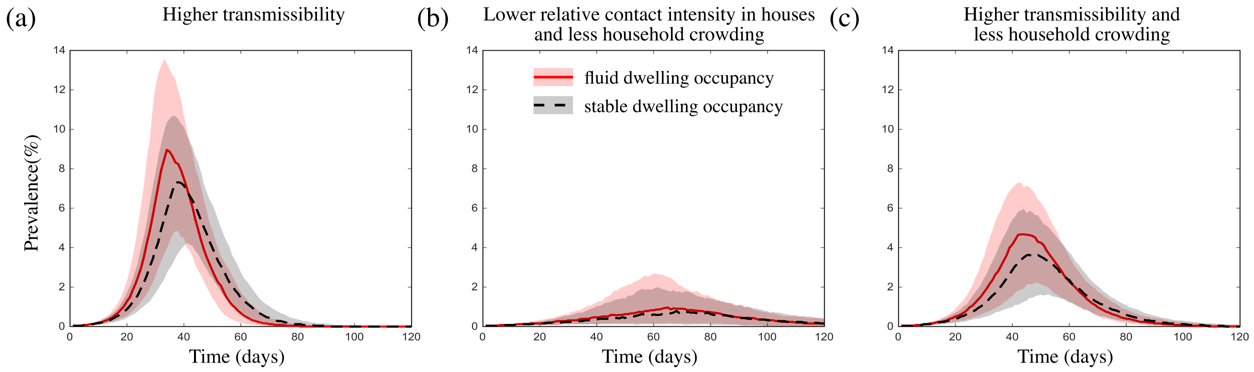

Figure S2: The impact of fluid dwelling occupancy on influenza-like outbreaks in a population of size $N = 2500$ assuming (a) a more transmissible pathogen; (b) a medium level of increased risk of transmission from household contacts compared to community contacts and less household crowding; and (c) a more transmissible pathogen and less household crowding. The lines and shading show the median and interquartile ranges of the population prevalence of infection over time when there is fluid dwelling occupancy (red solid line, red shading); compared to when there is stable dwelling occupancy (black dashed line and grey shading).

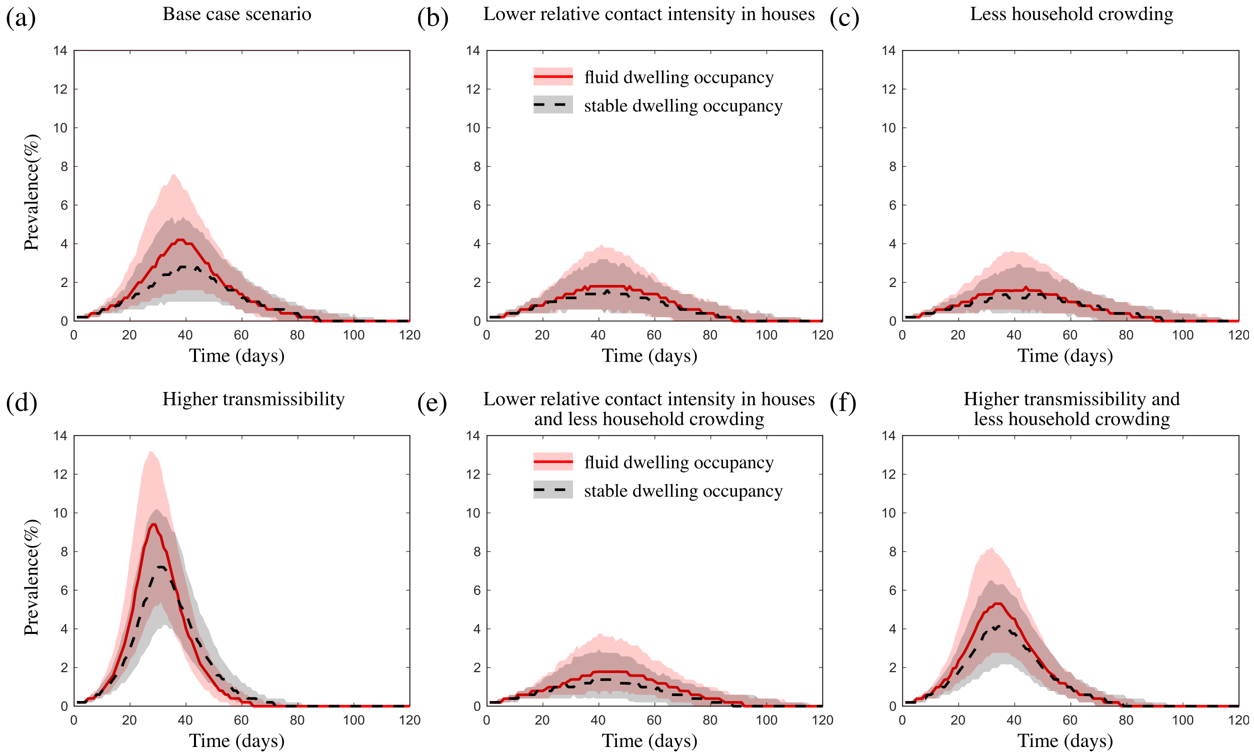

Figure S3: The impact of fluid dwelling occupancy on influenza-like outbreaks in a population of size $N = 500$ assuming (a) a high-level; and (b) a medium-level, of increased risk of transmission from household contacts compared to community contacts; (c) less crowding in dwellings; (d) a more transmissible pathogen; (e) a medium level of increased risk of transmission from household contacts compared to community contacts and less household crowding; and (f) a more transmissible pathogen and less household crowding. The lines and shading show the median and interquartile ranges of the population prevalence of infection over time when there is fluid dwelling occupancy (red solid line, red shading); compared to when there is stable dwelling occupancy (black dashed line and grey shading).

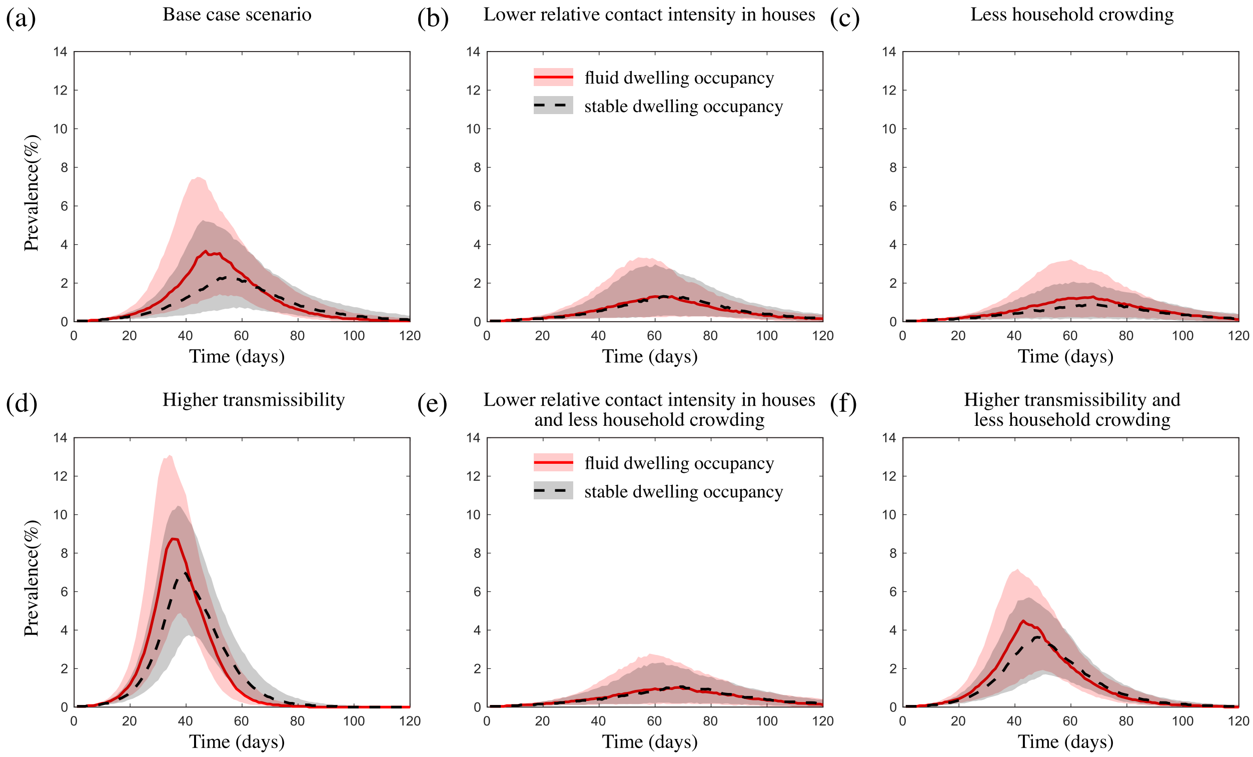

Figure S4: The impact of fluid dwelling occupancy with event-based migration on influenza-like outbreaks in a population of size $N = 2500$ assuming (a) a high-level; and (b) a medium-level, of increased risk of transmission from household contacts compared to community contacts; (c) less crowding in dwellings; (d) a more transmissible pathogen; (e) a medium level of increased risk of transmission from household contacts compared to community contacts and less household crowding; and (f) a more transmissible pathogen and less household crowding. The lines and shading show the median and interquartile ranges of the population prevalence of infection over time when there is fluid dwelling occupancy (red solid line, red shading); compared to when there is stable dwelling occupancy (black dashed line and grey shading).

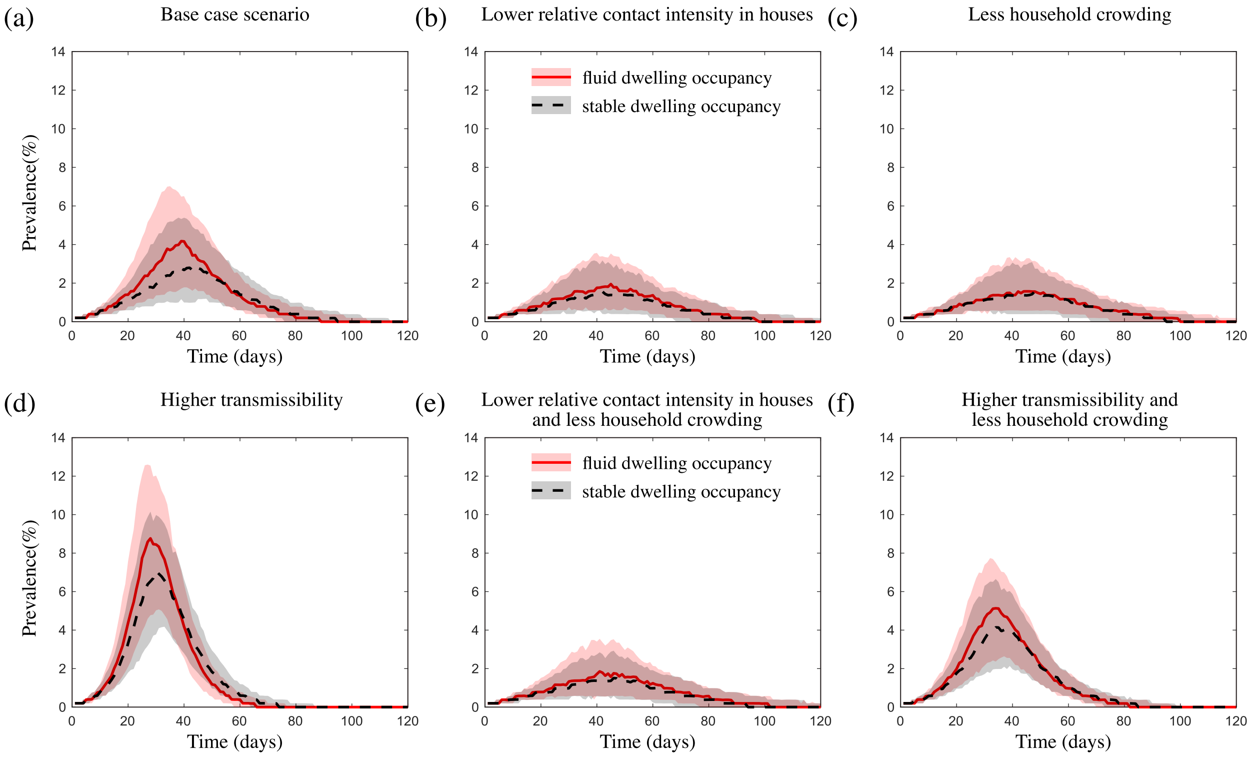

Figure S5: The impact of fluid dwelling occupancy with event-based migration on influenza-like outbreaks in a population of size $N = 500$ assuming (a) a high-level; and (b) a medium-level, of increased risk of transmission from household contacts compared to community contacts; (c) less crowding in dwellings; (d) a more transmissible pathogen; (e) a medium level of increased risk of transmission from household contacts compared to community contacts and less household crowding; and (f) a more transmissible pathogen and less household crowding. The lines and shading show the median and interquartile ranges of the population prevalence of infection over time when there is fluid dwelling occupancy (red solid line, red shading); compared to when there is stable dwelling occupancy (black dashed line and grey shading).

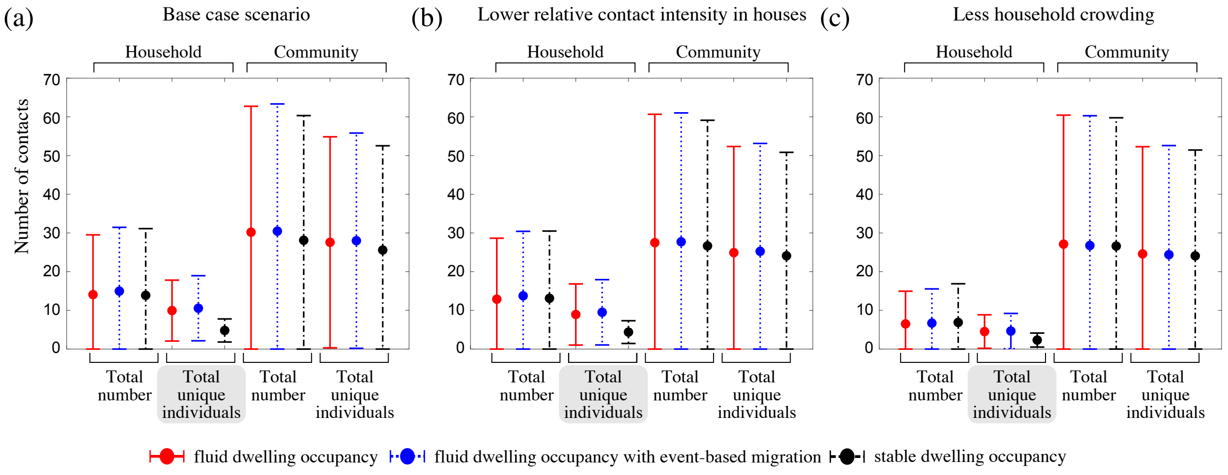

Figure S6: The impact of fluid dwelling occupancy on the distribution of the number of contacts of infectious people during outbreaks in a population of size $N = 500$ assuming (a) a high-level; (b) a medium-level, of increased risk of transmission from household contacts compared to community contacts; and (c) less crowding in dwellings. Each disk with error bars shows the mean of means $\pm$ one pooled standard deviation of either the total number of contacts, or the total number of unique individuals contacted (as indicated in the plots) during the infectious period of infected individuals, when there is fluid dwelling occupancy (red, solid lines); fluid dwelling occupancy with event-based migration (blue, dotted lines); compared to when there is stable dwelling occupancy (black, dash-dot lines).

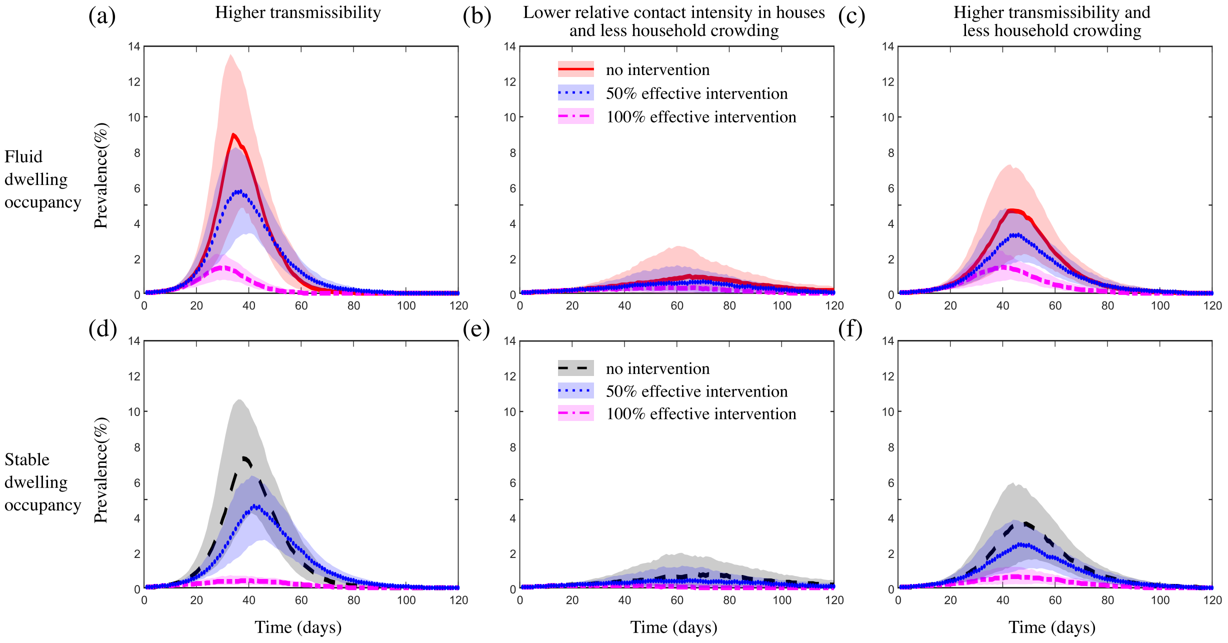

Figure S7: The impact of fluid dwelling occupancy on the effect of a household-focused prophylaxis intervention that is 50% effective and 100% effective in a population of size $N = 2500$ assuming (a,d) a more transmissible pathogen; and (b,e) a medium level of increased risk of transmission from household contacts compared to community contacts and less household crowding; and (c,f) a more transmissible pathogen and less household crowding. The lines and shading show the median and interquartile ranges of the population prevalence of infection over time when there is (a–c) fluid dwelling occupancy (unmitigated outbreak: red solid line and shading; with 50% effective intervention: blue dotted line and shading; with 100% effective intervention: magenta dash-dot line and shading); compared to when there is (d–f) stable dwelling occupancy (unmitigated outbreak: black dashed line and shading; with 50% effective intervention: blue dotted line and shading; with 100% effective intervention: magenta dash-dot line and shading)

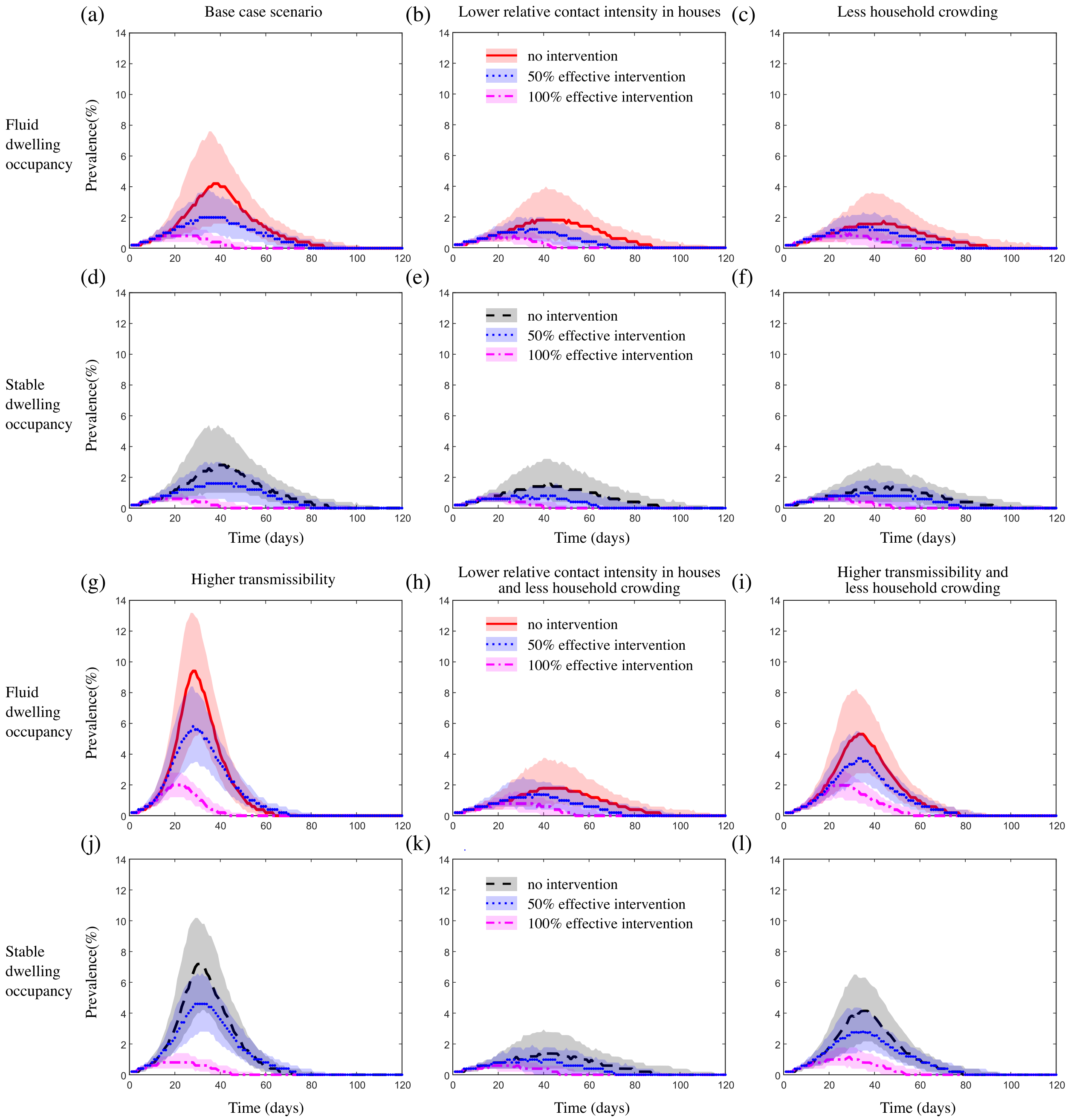

Figure S8: The impact of fluid dwelling occupancy on the effect of a household-focused prophylaxis intervention that is 50% effective and 100% effective in a population of size $N = 500$ assuming (a,d) a high-level; and (b,e) a medium-level, of increased risk of transmission from household contacts compared to community contacts; (c,f) less crowding in dwellings; (g,j) a more transmissible pathogen; and (h,k) a medium level of increased risk of transmission from household contacts compared to community contacts and less household crowding; and (i,l) a more transmissible pathogen and less household crowding. The lines and shading show the median and interquartile ranges of the population prevalence of infection over time when there is (a–c, g–i) fluid dwelling occupancy (unmitigated outbreak: red solid line and shading; with 50% effective intervention: blue dotted line and shading; with 100% effective intervention: magenta dash-dot line and shading); compared to when there is (d–f, j–l) stable dwelling occupancy (unmitigated outbreak: black dashed line and shading; with 50% effective intervention: blue dotted line and shading; with 100% effective intervention: magenta dash-dot line and shading)

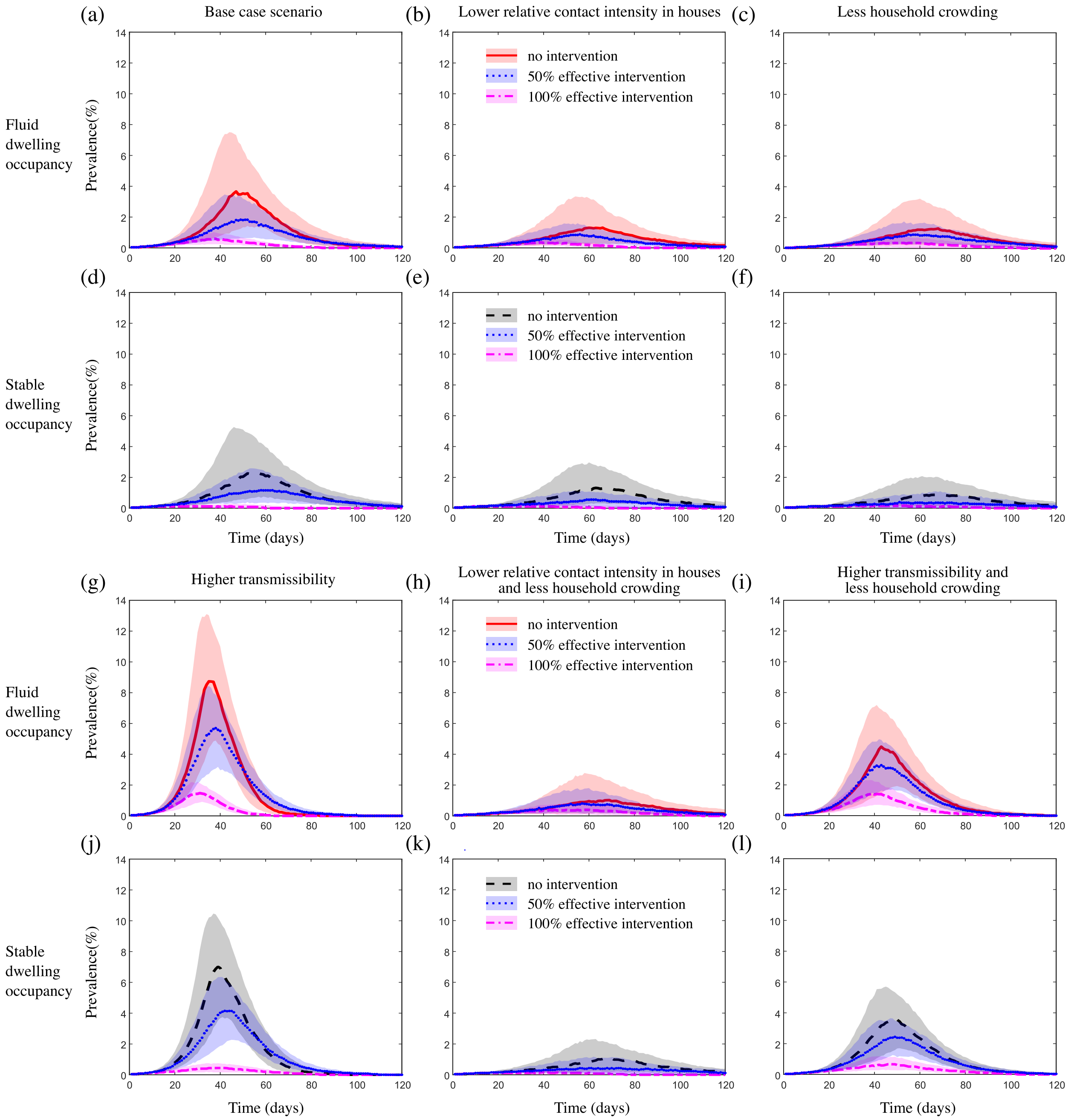

Figure S9: The impact of fluid dwelling occupancy with event-based migration on the effect of a household-focused prophylaxis intervention that is 50% effective and 100% effective in a population of size $N = 2500$ assuming (a,d) a high-level; and (b,e) a medium-level, of increased risk of transmission from household contacts compared to community contacts; (c,f) less crowding in dwellings; (g,j) a more transmissible pathogen; and (h,k) a medium level of increased risk of transmission from household contacts compared to community contacts and less household crowding; and (i,l) a more transmissible pathogen and less household crowding. The lines and shading show the median and interquartile ranges of the population prevalence of infection over time when there is (a–c, g–i) fluid dwelling occupancy (unmitigated outbreak: red solid line and shading; with 50% effective intervention: blue dotted line and shading; with 100% effective intervention: magenta dash-dot line and shading); compared to when there is (d–f, j–l) stable dwelling occupancy (unmitigated outbreak: black dashed line and shading; with 50% effective intervention: blue dotted line and shading; with 100% effective intervention: magenta dash-dot line and shading)

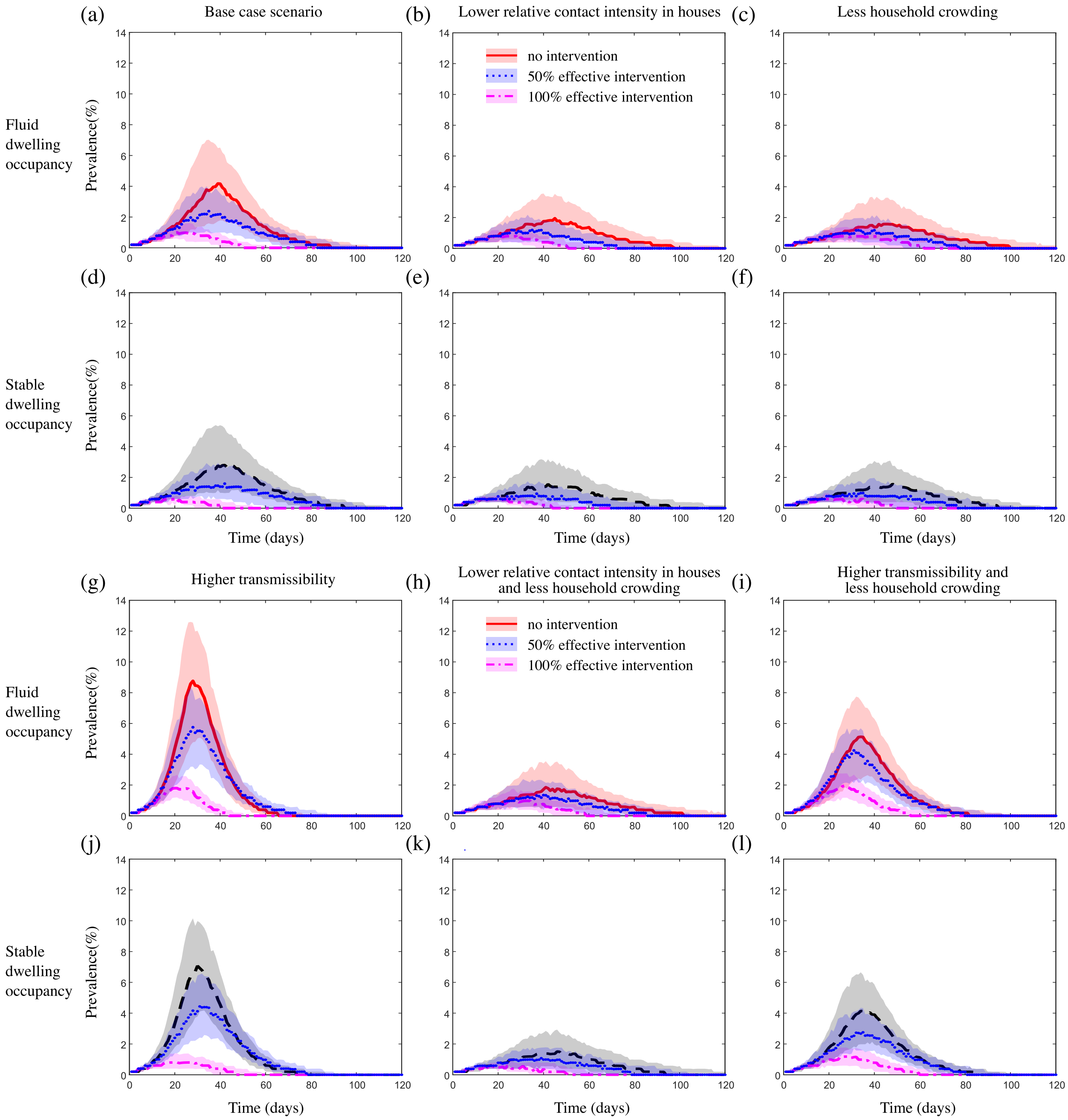

Figure S10: The impact of fluid dwelling occupancy with event-based migration on the effect of a household-focused prophylaxis intervention that is 50% effective and 100% effective in a population of size $N = 500$ assuming (a,d) a high-level; and (b,e) a medium-level, of increased risk of transmission from household contacts compared to community contacts; (c,f) less crowding in dwellings; (g,j) a more transmissible pathogen; and (h,k) a medium level of increased risk of transmission from household contacts compared to community contacts and less household crowding; and (i,l) a more transmissible pathogen and less household crowding. The lines and shading show the median and interquartile ranges of the population prevalence of infection over time when there is (a–c, g–i) fluid dwelling occupancy (unmitigated outbreak: red solid line and shading; with 50% effective intervention: blue dotted line and shading; with 100% effective intervention: magenta dash-dot line and shading); compared to when there is (d–f, j–l) stable dwelling occupancy (unmitigated outbreak: black dashed line and shading; with 50% effective intervention: blue dotted line and shading; with 100% effective intervention: magenta dash-dot line and shading)

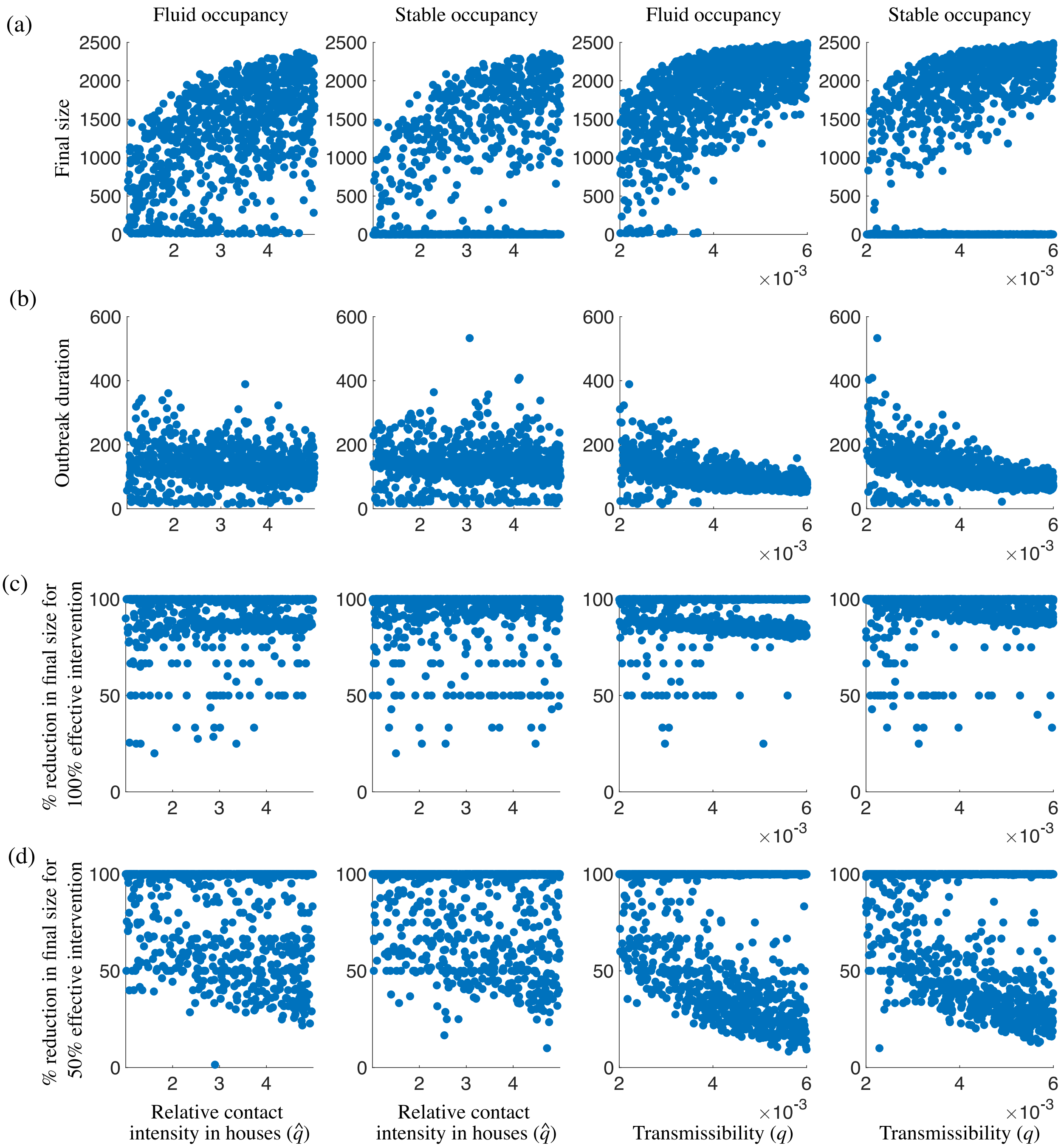

Figure S11: Parameter uncertainty analysis using Latin Hypercube Sampling (LHS). Correlation between the value of transmission parameters $\hat{q}$ (left two columns) and q (right two columns) and the model outcome variables (a) final size; (b) outbreak duration; (c) percentage reduction in final size from an intervention that is 100% effective; and (d) percentage reduction in final size from an intervention that is 50% effective. Here, $N = 2500$, $H = 358$, with fluid dwelling occupancy assumed (1st and 3rd columns) or stable dwelling occupancy assumed (2nd and 4th columns), and without event migration. Similar output for the other parameters sampled via LHS are not shown, but are briefly described here. The migration rate, α, showed no relationship with these outcome variables over the explored range of sampled values. Higher values of the duration of latency $1/\sigma$ were associated with a longer outbreak duration, but showed no relationship with other outcome variables. And higher values of the duration of infectiousness $1/\gamma$ were associated with a larger final size, a smaller reduction in final size due to both prophylaxis interventions, and showed no relationship with outbreak duration.
